# Sea Sickness

**Published:** 1869-02

**Authors:** 


*Sea Sickness.*—Dr. W. H. Dwinelle, of New York, has recently, on an Atlantic voyage, made a trial of Le Coniat's new method of treatment of this distressing accompaniment of travelling. Both he and his sister who was travelling with him, were very much prostrated, Dr. LeConiat made his application and in a few minutes gave entire relief. Though Dr. Dwinelle had been unable to keep any food on his stomach from Saturday afternoon till Tuesday morning, he was able to rise and eat his breakfast. In a number of cases, ladies who had been unable to lift their heads from the pillow for several days, were actually relieved so that they got out of bed and took their seats at the dinner-table.

The treatment consists in laying the patient horizontally, uncovering the region of the stomach, and applying to the skin a solution of one grain of sulphate of atropia in an ounce of water. He then sets a flat disc connected with the negative pole of a galvanic battery, over the pyloric orifice of the stomach, and passes a nerisl sponge attached to the positive pole over the surface from the cardiac to the pyloric orifice, keeping up the manipulations four or five minutes and varying them by occasional vertical passes downward. The muscles are seen to contract vigorously during the operation. The theory is that the vomiting of sea-sickness depends upon an inverted peristaltic action of the stomach, and that these manipulations restore the normal motion. He says he cures 90 per cent. of his patients, and that the same treatment is applicable to the vomiting of pregnancy.

"The battery used by Dr. Le Coniat is one of the ordinary vibrating, carbon and amalgamated zinc order, capable of double gradation. The solution for bathing is made as follows: Take ½ oz. of bicarbonate of potash, dissolved in 9 oz of warm water; when cold, add ½ oz. of sulphuric acid."

